# Vestibular dysfunction: a hidden risk factor for Alzheimer’s disease

**DOI:** 10.1007/s00415-025-13045-9

**Published:** 2025-03-25

**Authors:** Carolin Anna Maria Koriath, Boris Rauchmann, Florian Schoeberl, Andreas Zwergal, Peter Falkai, Robert Perneczky

**Affiliations:** 1 Department of Psychiatry and Psychotherapy, LMU University Hospital, LMU Munich, Munich, Germany; 2 Department of Neurology, LMU University Hospital, LMU Munich, Munich, Germany; 3 German Center for Vertigo and Balance Disorders (DSGZ), LMU University Hospital, LMU Munich, Munich, Germany

**Keywords:** Alzheimer’s disease, Dementia, Vertigo, Sensory systems, Neurodegeneration, Aging

## Abstract

**Background:**

London taxi drivers’ navigationally challenged hippocampi are known to be enlarged, and reduced Alzheimer’s disease (AD)-related mortality has recently been shown in similarly well-versed drivers, implying a neuroprotective effect through hippocampal engagement. Vestibular function has been linked to hippocampal size, suggesting that vestibular input may influence AD risk.

**Methods:**

Including 16 known modifiable lifestyle factors as covariates, we analyzed UK Biobank (UKB) volunteers aged over 55 years and without dementia at baseline to assess how peripheral vestibular dysfunction (PVD) influences the likelihood of an AD diagnosis.

**Results:**

4684 AD and 2133 PVD cases were identified based on their ICD diagnoses; even accounting for other risk factors, PVD increased the risk of AD 1.7 times in UKB volunteers.

**Discussion:**

Vestibular loss, linked to hippocampal atrophy and default mode network disruption, appears to increase AD risk. Consequently, active vestibular stimulation by balance training or neuromodulation could offer potential for modifying AD progression.

## Introduction

In an aging society, interest and research in dementia have been expanding over recent decades as its increasing prevalence is placing an ever heavier burden on care systems and the population [1]. While research focused on familial forms of early-onset dementia with Mendelian inheritance (such as familial Alzheimer’s disease (AD), familial frontotemporal dementia (FTD) or inherited prion disease) has been crucial to furthering our understanding of the underlying clinical-pathological processes [2], in older age, most cases appear to be sporadic, with AD the commonest late-onset dementia [3]. A recent study showed reduced AD-related mortality among drivers frequently engaged in navigational tasks, suggesting a neuroprotective effect of spatial cognition and hippocampal engagement [4]. AD patients have been demonstrated to suffer from balance and vestibular function impairment [5, 6], and falls in the otherwise healthy older adults have been shown to predict future dementia diagnoses [7], underscoring the links between the vestibular system and AD dementia, although a causal link has never been proven. Previously, the posterior hippocampi of highly trained navigator London taxi drivers have been shown to be significantly larger relative to those of control subjects, while similarly, the hypertrophied posterior hippocampi of ballet dancers and slackliners demonstrated the effects of intensive balance training [8]. Conversely, patients with acquired chronic bilateral vestibular loss develop atrophy of the hippocampus [9], impacting spatial memory [10, 11]. These patients show selective deficits in finding novel routes in real space, alongside reduced navigation-induced right hippocampal activation, suggesting that allocentric spatial orientation relies on vestibular input [12, 13]. Based on these findings, it appears possible that vestibular sensory input may influence the resilience of cerebral networks and thereby the risk of dementia [14–16]; we, therefore, asked whether the elderly with peripheral vestibular dysfunction are at a higher risk of AD.

## Methods

In order to test this hypothesis, we analyzed data from the UK Biobank (UKB), a population-based, deeply phenotyped cohort of more than 500,000 participants aged 40–69 years at recruitment linked to their continually updated health records [17]. Ethics approval was provided by the National Information Governance Board for Health and Social Care and the National Health Service North West Multicentre Research Ethics Committee; all participants provided informed consent through electronic signature at the baseline assessment.

After downloading and unpacking the data in R (version 2024.04.1 + 748) using ukbtools [18], the dataset of 502,414 participants was filtered to only include participants aged over 55 years of age at baseline to allow time for participants to develop late-onset AD during the 17-year follow-up between recruitment and data dispensation; participants with dementia known to have been diagnosed prior to their first assessment were excluded using the UKB variable “date_of_all_cause_dementia_report_f42018_0_0”. A logistic regression was run using a glm model in R to calculate the impact of peripheral vestibular dysfunction on the likelihood of an AD diagnosis, including known modifiable lifestyle factors as covariates [19]. Other forms of dementia, central vertigo, and unspecified dizziness were excluded from the analysis. To create the relevant covariate variables, subsets of participants were created for AD, hearing loss, obesity, hypertension, and depression by extracting ICD9 and ICD10 diagnoses from the dataset. For the subset “vestibular dysfunction”, this included the ICD10 codes “H81.0 Menière disease”, “H81.1 Benign paroxysmal vertigo”, “H81.2. Vestibular neuronitis”, “H81.3 Other peripheral vertigo”, “H81.8 Other disorders of vestibular function”, “H81.9 Disorders of vestibular function, unspecified”, but excluded “H81.4 Vertigo of central origin” to focus on peripheral sensory input. For ICD9, the diagnoses “386.1 Vestibular neuronitis”, “386.2 Benign paroxysmal positional vertigo (BPPV)”, “386.3 Labyrinthitis”, “386.5 Labyrinthine dysfunction”, “386.8 Other specified disorders of vestibular function”, and “386.9 Unspecified vertiginous syndrome” were included in the vestibular dysfunction subset, while “386.4 Vertigo of central origin” was excluded. UKB data variables were coded as factors for the Townsend deprivation index at recruitment, excess alcohol consumption (> 6 units of alcohol at least weekly), physical activity (1–2 h of moderate exercise), age when full time education was completed (as a measure of overall education), inverse distance to the nearest major road (as a measure of air pollution), diabetes, loneliness and isolation, sleeplessness and insomnia, pack years of smoking, and whether someone used a hearing aid. Since both AD and vestibular disorders have a strong relationship with age, in addition to age at first assessment, we added age^2^ and age^3^ terms into the model to account for a likely non-linear relationship. We also filtered the data based on the encoded dates of the ICD diagnoses to exclude participants diagnosed with AD prior to a diagnosis of peripheral vestibular dysfunction (PVD).

## Results

Baseline characteristics of participants are shown in Table 1. Filtering the 502,414 UKB participants to only include participants aged over 55 years of age at baseline left 291,426; after excluding participants with dementia known to have been diagnosed prior to their first assessment, 291,240 participants were included in the analysis. Having already removed all participants who were diagnosed with any type of dementia prior to their first UK Biobank assessment (using the “date of all dementia report” variable), we found that the ICD diagnoses for both PVD and AD were coded on the same day for all cases diagnosed with both. Using the “date of all dementia report” variable again, we found that, where this variable had been encoded, the date of the dementia report was later than the ICD PVD diagnosis dates in all cases where both diagnoses were present. We, therefore, proceeded with the analysis. Of the 291,240 participants, 155,871 were female and 135,369 were male. According to their health records, 4684 participants were diagnosed with AD after their initial UKB assessment, or their date of diagnosis was censored, and 2133 participants had been diagnosed with peripheral vestibular disorders. Furthermore, 16,373 participants had a history of depression, 19,341 participants had been diagnosed with diabetes by a doctor, 21,268 participants had been diagnosed as obese, 1750 participants had had a traumatic brain injury (TBI), 9,742 participants had been diagnosed with hearing loss and 113,586 suffered from hypertension. Based on their questionnaire answers, 15,955 participants drank > 6 units of alcohol at least monthly, 12,030 participants reported at least 1–2 h of moderate exercise, 47,897 participants reported feelings of loneliness, 228,528 participants reported insomnia at least sometimes, and 12,676 participants used a hearing aid. Unavailable data for these variables was interpreted as a negative response. Participants completed their full time education at a mean of 16.16 years, were on average 62.47 years old at their first UKB assessment, had a mean of 25.75 pack years of smoking tobacco, and a mean Townsend index of deprivation of – 1.5102. Participants mean inverse distance to the next major road was 0.005655754 (see Table 1).

**Table 1:** Overview of the characteristics of the cohort

Characteristic	N of 291,240 participants
UKB participants > 55 years without dementia old at first assessment	291,240
Female sex	155,871
Participants with vestibular dysfunction	2133
Participants diagnosed with AD	4684
Participants diagnosed with hearing loss	9742
Participants diagnosed with TBI	1750
Participants diagnosed with obesity	21,268
Participants diagnosed with hypertension	113,586
Participants diagnosed with depression	16,373
Participants diagnosed with diabetes	19,341
Participants drinking > 6 units of alcohol at least weekly	10,285
Participants reporting moderate exercise at least 1–2 h	12,030
Participants reporting frequent feelings of loneliness	47,897
Participants reporting frequent insomnia	228,528
Participants using a hearing aid	12,676
Average Townsend index of deprivation	– 1.5102
Average age at leaving full time education	16.16 years
Average inverse distance to the next major road	0.005655754
Average age at first assessment	62.47 years
Average pack years of smoking tobacco	25.75 years

The total of 502,414 UKB participants were filtered to only include those over 55 years old at the first assessment, leaving 291,426 participants. From this cohort all those with a diagnosis of any dementia before their first assessment were removed, leaving 291,240 participants in the study cohort

*AD* Alzheimer’s disease, *TBI* traumatic brain injury, *N* number

**Image 1:**
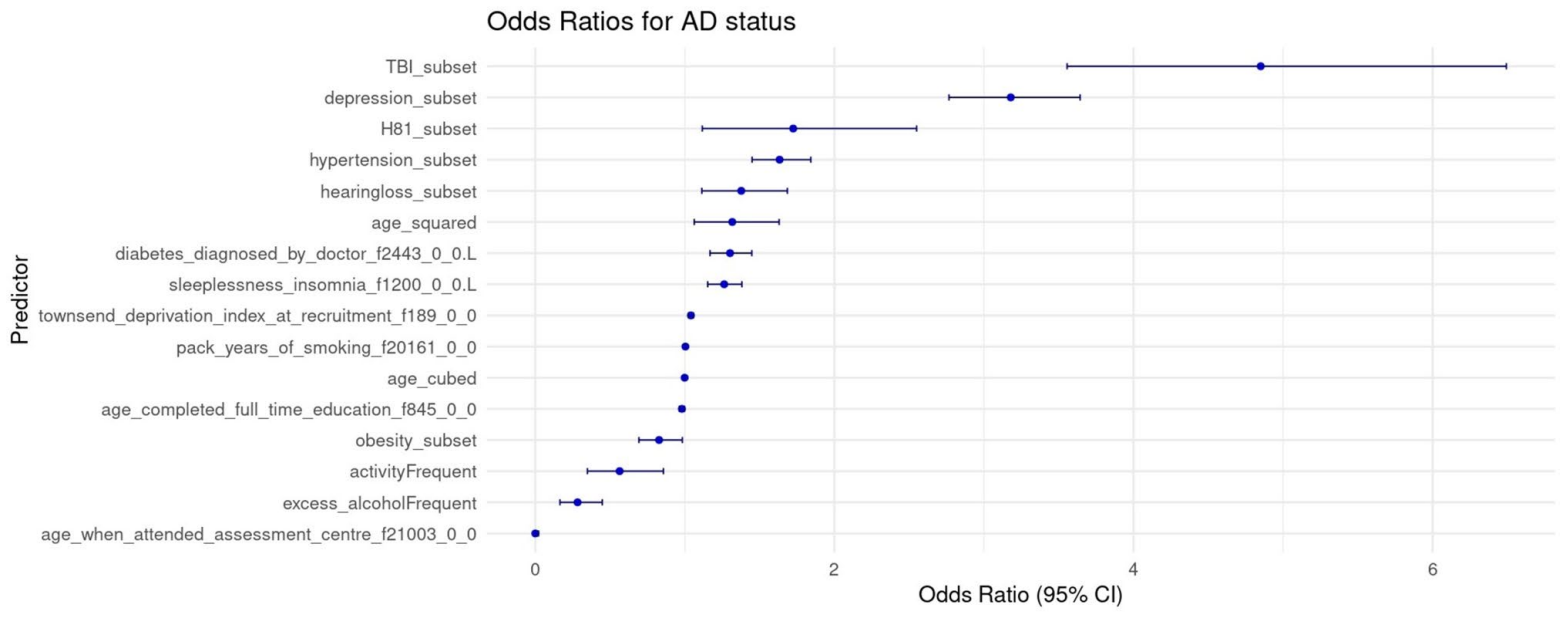
Odds ratios and 95% confidence intervals for the prediction of Alzheimer’s disease. Shown are odds ratios for Alzheimer’s disease (AD) with their 95% confidence intervals (x-axis) for each significant predictor in the analysis (y-axis). *AD* Alzheimer’s disease, *TBI* traumatic brain injury, *H81_subset* vestibular dysfunction

The logistic regression showed that hearing aid use, male or female sex, loneliness, and the inverse distance to a major road were not significantly correlated with a diagnosis of AD in our analysis and were, therefore, removed as covariates. In our analysis, UKB volunteers with vestibular dysfunction were 1.7 times as likely to receive a diagnosis of AD as those without (*p* = 0.0094, OR = 1.72, CI 1.12–2.55), even accounting for other contributing covariates. Other significant factors increasing the likelihood of AD included the Townsend deprivation index at recruitment (*p* = 1.97e-06, OR = 1.04, CI 1.02–1.06), hearing loss (*p* = 0.0024, OR = 1.38, CI 1.11–1.69), traumatic brain injury (TBI) (*p* < 2e-16, OR 4.85, CI 3.56–6.492), hypertension (*p* = 9.91e-16, OR = 1.63, CI 1.45–1.84), depression (*p*: 2e-16, OR = 3.18, CI 2.77–3.64), age (*p*, OR = 2.68 e-8, CI 3.69 e-14–0.0212), age^2^ (*p*: 0.0016, OR = 1.32, CI 1.06–1.63), age^3^ (p: 0.0127, OR = 0.999, CI 0.997–1.00), sleeplessness/insomnia (*p* = 4.19e-07, OR = 1.26, CI 1.15–1.38), and diabetes (*p* = 1.40e-06, OR 1.30, CI 1.17–1.45). In our analysis, frequent (at least monthly) consumption of more than 6 units of alcohol significantly decreased the likelihood of AD (*p* = 6.02e-05, OR 0.283, CI 0.165–0.448), as did obesity (*p* = 0.0342, OR = 0.83, CI 0.69–0.98), frequent moderate physical activity (*p* = 0.012000 OR = 0.56, CI 0.35–0.86), and education (*p* = 0.024, OR = 0.98, CI 0.96–0.998), while pack years of smoking only had a marginal effect (*p* = 0.0009, OR = 1.00, CI 1.00–1.01). An interaction analysis between vestibular dysfunction and hearing loss showed no significant interaction. See Image 1 and Table 2 for details.

**Table 2:** Odds ratios and confidence intervals

Covariates	Odds_Ratio	CI_Lower	CI_Upper	Pr( >|z|)
(Intercept)	1.18E + 156	8.24E + 31	1.78E + 279	0.013336*
H81 subset	1.72	1.12	2.55	0.009387**
Townsend deprivation index at recruitment	1.04	1.02	1.06	1.97e-06***
Hearing loss subset	1.38	1.11	1.69	0.002448**
TBI subset	4.85	3.56	6.49	< 2e-16***
Obesity subset	0.828	0.693	0.983	0.034185*
Hypertension subset	1.63	1.45	1.84	9.91e-16***
Depression subset	3.18	2.77	3.64	< 2e-16***
Excess alcohol frequent	0.283	0.165	0.448	6.02e-07***
Activity frequent	0.564	0.349	0.857	0.011997*
Age completed full time education	0.980	0.963	0.998	0.024449*
Age when attended assessment center	0.0268E-02	0.000369	0.0212E	0.011565*
Age squared	1.32	1.06	1.63	0.011671*
Age cubed	0.999	0.997	1.00	0.012662*
Diabetes diagnosed by doctor	1.30	1.17	1.45	1.40e-06***
Sleeplessness insomnia	1.26	1.15	1.38	4.19e-07***
Pack years of smoking	1.00	1.00	1.01	0.000899***

Many known modifiable risk factors for Alzheimer’s disease (AD) were highly significant and had strong effect sizes; still vestibular dysfunction increased the likelihood of an AD diagnosis strongly and significantly

*AD* Alzheimer’s disease, *TBI* traumatic brain injury, *CI* confidence interval. Level of significance: * = <0.05, ** = <0.01, *** = <0.001

## Discussion

Patients with AD have been known to suffer from higher rates of vestibular dysfunction for some time [20]; a recent study has shown a dose–response relationship between cognitive decline and vestibular dysfunction [21]. So far, whether this is due to cause or effect has been widely contested, with some suggesting that that early neurodegenerative change might either cause vestibular symptoms or increase the likelihood of seeking primary care for vestibular disorders. However, new evidence now points towards a protective effect of intense navigational training [4], suggesting that vestibular deficits may indeed be an independent risk factor for AD. Focusing on a population-based approach, our analysis demonstrates that UKB volunteers with vestibular dysfunction are 1.7 times as likely to be diagnosed with AD even taking into account other known risk factors.

Vestibular function [22] and functional vestibular cortical connectivity [23] demonstrably decline with age; clinically relevant peripheral vestibular dysfunction is most likely underdiagnosed given a prevalence of balance dysfunction of 35% even in relatively young adults [24], long before early neurodegenerative changes would be expected even in those patients who later go on to develop AD or other dementias. Falls in otherwise healthy older adults can predict future dementia diagnoses [7], likely reflecting neuron loss in cholinergic and aminergic nuclei [25]. However, this more likely follows vestibular input loss, rather than causing peripheral sensory loss. Non-specific dizziness and falls often stem from autonomic dysfunction or gait disturbances, e.g. due to Parkinson’s disease dementia [26] or later stages of dementia, particularly in care homes [27–29]. While patients with clinical dementia exhibit higher healthcare utilization, including more frequent primary care visits, hospitalizations, and prescriptions [30], evidence suggests that this is primarily due to complex medical needs, such as fractures, cardiovascular diseases and neuropsychiatric symptoms [31], with utilization increasing over time. It is, therefore, crucial to distinguish peripheral vestibular deficits as a distinct sensory dysfunction from non-specific dizziness and falls, which may signal advancing neurodegenerative disease. For this reason, we excluded central vestibular deficits and dizziness/giddiness from our analysis. Peripheral vestibular dysfunction as a specific risk factor for AD should lead to more accurate population screening in middle age and updates to the clinical care of patients with vestibular disorders. In addition, it raises the question whether regular balance training may delay the progression of mild cognitive impairment to the full clinical picture of dementia as new evidence now points towards a protective effect of intense navigational training [4]. As expected, education and moderate physical activity slightly decreased the likelihood of an AD diagnosis, possibly indicating the strength of underlying connections, networks and training effects. While (at least monthly) consumption of more than 6 units of alcohol and obesity decreased the likelihood of an AD diagnosis, sex and hearing aid use did not have a significant effect. For obesity, this may be due to our cohort’s mean age of 62, where the harmful effects in middle age transition to frailty protection in later life [32].Our results are consistent with the neurobiology of the central vestibular system [33–35] and with previous evidence that acquired chronic bilateral vestibular loss leads to hippocampal atrophy [9, 10], and that unilateral vestibular loss disrupts the default mode network [36]. While vestibular dysfunction earlier in life has been shown to increase the risk of AD [37], PVD appears not to be directly associated with beta-amyloid deposition in AD patients or to directly influence this aspect of the pathology [38]. One possible explanation may be that, in AD, the posterior default mode network fails before amyloid plaques become measurable; this initiates a connectivity cascade involving hubs of high connectivity, which in turn are associated with amyloid accumulation [39]. The influence of modifiable risk factors, such as vestibular function, depression, and TBI, on network disruption may go some way to explain the gap between neuropathology and clinical deficits. Beyond their impact on progression, disorders of vestibular function may even influence the development of AD sub-phenotypes with a predominant affection of visuospatial cognitive domains [40, 41], and to be markers of premature aging and harbingers of dementia [14]. Inversely, impairment of allocentric spatial orientation performance is a robust predictor of AD pathology in patients with mild cognitive impairment [42]. Frequent practice of navigational tasks, balance training, and potentially targeted vestibular stimulation may therefore foster hippocampal resilience with potentially disease modifying effects. Recently, non-invasive noisy galvanic stimulation of primary vestibular afferents has been shown to improve spatial cognition in animal models of vestibular loss [43] and cognitive impairment [44]. Limitations of this study include the lack of diagnosis timing for all cases of PVD and AD, the correlational nature of the findings, and the underdiagnosis of PVD, meaning some participants may not have an ICD-recorded diagnosis. Further research is therefore needed to provide a deeper understanding of how vestibular function may impact cerebral networks and their degeneration.

## Data Availability

This research was conducted using data from the UK Biobank under application number 69623. The UK Biobank data are available to bona fide researchers upon application and approval. Further information about the access process is available at https://www.ukbiobank.ac.uk/enable-your-research.
